# The measurement of alexithymia in children and adolescents: Psychometric properties of the Alexithymia Questionnaire for Children and the twenty-item Toronto Alexithymia Scale in different non-clinical and clinical samples of children and adolescents

**DOI:** 10.1371/journal.pone.0177982

**Published:** 2017-05-25

**Authors:** Gwenolé Loas, Stéphanie Braun, Marie Delhaye, Paul Linkowski

**Affiliations:** Department of Psychiatry & Laboratory of Psychiatric Research (ULB 266), Cliniques universitaires de Bruxelles, Université Libre de Bruxelles (ULB), Bruxelles, Belgium; Radboud Universiteit, NETHERLANDS

## Abstract

This study had two aims. Firstly, the psychometric properties of the 20-item Toronto Alexithymia Scale (TAS-20) and the Alexithymia Questionnaire for Children (AQC) that measure the three dimensions of alexithymia (DIF, difficulty identifying feelings; DDF, difficulty describing feelings; EOT, externally-oriented thinking) were explored in various samples of children, adolescents or young adults to detect the best factor-structure and to examine if the Externally-Oriented Thinking (EOT) factor must be deleted or not. Secondly, the capacity for adolescents to distinguish between alexithymia and depression was studied using factorial analyses of items of self-report of alexithymia and depression scales. Four groups were examined (80 healthy children, 105 adolescents with various psychiatric disorders, 333 healthy older adolescents and 505 young adults recruited from universities). The first two groups filled out the AQC and the latter two the TAS-20. Confirmatory factorial analyses (CFA) showed that the two-factor model (DIF, DDF) provided acceptable fits and had significant advantages over the three-factor model (DIF, DDF, EOT). Low alpha coefficients for the EOT subscale were reported (range from 0.18–0.61). Except for the children sample, exploratory factorial analyses (EFA) were performed on the items of the TAS-20 or AQC without the EOT items and the Beck depression inventory-II (BDI-II) or the Zung Self-Rating Depression Scale (SDS). The items of the AQC and BDI-II or items of the TAS-20 and SDS loaded on separate factors with only a minor overlap suggesting that adolescents were able to differentiate alexithymia and depression when self-assessments were used. Alexithymia can be reliably assessed in adolescents using the TAS-20 or AQC without the eight items rating the EOT dimension.

## Introduction

Alexithymia is not a diagnostic category included in any mental disorder but a multifaceted personality construct associated with various somatic or psychiatric disorders as well as nonclinical populations [1]. This personality construct is characterized by four main features: 1) difficulty identifying and distinguishing emotions from bodily sensations; 2) difficulty describing and verbalizing emotions; 3) poverty of fantasy life; 4) externally oriented thinking. One of the main reasons for studying alexithymia is its heuristic value. For example one recent review proposed expanding the concept of alexithymia to that of affective agnosia [2].

Although research with children or adolescents is relatively limited, several studies suggest that alexithymia could have the same consequences for health in infancy or adolescence as in adulthood. Indeed, affect regulation and the quality of attachment are closely related and the exploration of alexithymia in adolescence or childhood and notably its relationships with the attachment style gives us the opportunity to examine the capacity to regulate emotion independently of reliance on the primary caregiver. It would seem that alexithymia in adolescents moderated, and partially mediated, the relationship between bullying and deliberate self-harm [3]. In female adolescents, multivariate logistic regression analyses indicated that only alexithymia (and not dissociation or childhood maltreatment) was a significant predictor for non-suicidal self-injury [4].

One of the main limitations of the exploration of alexithymia in adolescence or infancy is the use of adult measures for assessing the construct. Almost all of the existing adolescent alexithymia research studies have been conducted with the twenty-item Toronto Alexithymia Scale (TAS-20, [5]), the most widely used measure of alexithymia. The TAS-20 comprises three subscales: (1) Difficulty Identifying Feelings (DIF); (2) Difficulty describing feelings (DDF); (3) Externally-oriented thinking (EOT). An adapted version of the TAS-20 for use in adolescents or children (the Alexithymia Questionnaire for Children, AQC) has been developed by Rieffe et al. [6] but is, unfortunately, seldom used.

Nine studies have explored the psychometric properties of the TAS-20 in children or adolescents [7–15] and two other studies have explored the validity and the reliability of the AQC [8, 16]. Eight out of the eleven aforementioned studies used confirmatory factorial analyses (CFA) first to test the original three-factor model of the scales and then Cronbach’s alpha coefficient to measure the reliability of the scales. (Table 1).

**Table 1:** Psychometric properties of the TAS-20 or AQC in children or adolescents.

Authors	Samples	Age	Origin	CFA	α
Rieffe et al, 2006	740 children (380 boys, 360 girls)	Mean age 12.4 years, range: 9.6–15.1	Primary and secondary schools	Three-factor (DIF, DDF, EOT)	**0.73** (DIF) **0.75** (DDF) 0.29 (EOT) ? (AQC)
Joukamaa et al, 2007	6000 girls and boys	Range:15–16 years	Community	Three-factor (DIF, DDF, EOT)	Not precised
Säkkinen et al, 2007	882 (478 boys, 404 girls)	Mean age 14.6 years, range: 12–17	Secondary schools	Three-factor (DIF, DDF, EOT)	**0.78** (DIF) 0.64(DDF) 0.57 (EOT) **0.73** (TAS-20)
Zimmermann et al, 2007	264 (155 boys, 109 girls)	Mean age 16.53 years, range:14–19	Secondary, professional schools	Three-factor (DIF, DDF, EOT)	0.66 (DIF) **0.71**(DDF) 0.43 (EOT) ? (TAS-20)
Karukivi et al, 2010	729 (539 girls, 190 boys)	Mean age 19 years, range: 17–21	Students or other	Three-factor (DIF, DDF, EOT)	Not precised
Loas et al, 2010	80 (43 boys, 37 girls)	Mean age 11.8 years, range: 9–16	Community	Three-factor (DIF, DDF, EOT)	0.56 (DIF) 0.66 (DDF) 0.29 (EOT) 0.64 (AQC)
Parker et al, 2010	267 (109 boys, 158 girls)	Range: 19–21 years	Community	Three-factor (DIF, DDF, EOT)	**0.80** (DIF) **0.79** (DDF) **0.74** (EOT) **0.87** (TAS-20)
	288 (103 boys, 185 girls)	Range: 17–18 years	Community	Three-factor (DIF, DDF, EOT)	**0.74** (DIF) **0.81** (DDF) 0.68 (EOT) **0.82** (TAS-20)
	297 (119 boys, 178 girls)	Range: 15–16 years	Community	Three-factor (DIF, DDF, EOT)	0.66 (DIF) **0.80** (DDF) 0.52 (EOT) **0.75** (TAS-20)
	149 (80 boys, 69 girls)	Range: 13–14 years	Community	Three-factor (DIF, DDF, EOT)	0.52 (DIF) 0.67 (DDF) 0.49 (EOT) 0.68 (TAS-20)
Meganck et al, 2012	406 (187 boys, 219 girls)	Range: 12–17 years	Secondary schools	Three-factor (DIF, DDF, EOT) Four-factor (DIF, DDF, PR, IM)	**0.80** (DIF) 0.67 (DDF) 0.43 (EOT) 0.10 (PR) 0.44 (IM) **0.72** (TAS-20)
Loas et al, 2012	140 (18 boys, 122 girls)	Mean age 17.3 years, range: 15–18	Borderline personality (59) and secondary schools (81)	Three-factor (DIF, DDF, EOT)	**0.84**(DIF) **0.77** (DDF) 0.53 (EOT) **0.80** (TAS-20)
Craparo et al, 2015	508 (248 boys, 260 girls)	Mean age 12.56 years, range: 12–13	Not precised	Two-factor (DIF, DDF)	Not precised
Ling et al, 2016	1260 (641 boys, 619 girls)	Mean age 14.62 years, range: 12–18	Middle schools	Four-factor (DIF, DDF, PR, IM)	**0.84** (DIF) **0.77** (DDF) 0.47 (PR) 0.47 (IM) **0.87** (TAS-20)

In boldface Cronbach’s α ≥ 0.7;

TAS-20: 20-item Toronto Alexithymia Scale, AQC: Alexithymia Questionnaire for Children, DIF: difficulty identifying feelings subscale of the TAS-20; DDF: difficulty describing feelings of the TAS-20, EOT: externally-oriented thinking of the TAS-20; PR: pragmatic thinking of the TAS-20, IM: lack of subjective significance or importance of emotions of the TAS-20.

Eight of these studies found that the original three-factor model provided acceptable fit and three reported that a four-factor model provided significant better fits than the three-factor model [12, 15] and that a two-factor model (DIF and DDF) [14] provided acceptable fit. In the four-factor model EOT split into two factors “pragmatic thinking” (PR, items 5, 8, 20) and “lack of subjective significance or importance of emotions” (IM, items 10, 15, 16, 18, 19). Regarding the reliability of the scale or its subscales, all of the studies reported low reliability of the EOT subscale with values ranging from 0.74 to 0.29. It is important to note that the reliability of the EOT subscale decreases with age (Table 1).

Taking into account the low reliability of the EOT subscale in adolescents or children, one study [17] with 796 students (modal age of 13 years), has explored their ability to discriminate between their own emotional states when using the TAS-20 without the EOT items and their levels of self-esteem and trait-hope when using self-report scales. The Cronbach alpha for the twelve items of the TAS-20 was 0.87. Using exploratory factor analysis on the 12 items of the TAS-20, the 10 items of the Rosenberg self-esteem scale and the 6 items of the children‘s hope scale, a three-factor solution was found, the three dimensions loaded on separate factors without overlap. However, another exploratory factorial analysis was performed using the 12 items of the TAS-20 and the Positive and Negative Affect Schedule to explore the ability of adolescents to discriminate alexithymia from negative and positive affectivity. A five-factor solution emerged with an alexithymia factor and four affective factors without overlap of the alexithymia items on the affective factors. Thus, the authors concluded that, when only using 12 items of the TAS-20, alexithymia is reliably and validly measured in adolescents and is distinguishable from self-esteem, hope, positive and negative dimensions.

Another study [18] has assessed alexithymia in Japanese adolescents using the TAS-20. 202 students (102 boys, 100 girls) with a mean age of 13.86 years (sd = 0.95, range: 12–15) completed the Japanese version of the TAS-20 that was linguistically modified to make it more comprehensible for adolescents. Moreover, the authors added nine new items: three to measure EOT, six to measure reduced capacity for imagination (RCI). An EFA was performed and a four-factor solution was retained with 13 items having factor loadings higher than 0.4. DIF, DDF, EOT and RCI had 4, 2, 4 and 3 items, respectively. Among the 13 items only 7 (4 DIF, 2 DDF, 1 EOT) were items from the original scale. the authors conducted a CFA to validate the four-factor structure of the EFA. The four-factor model had acceptable fits and all factor loadings were significant. The authors concluded that alexithymia in early adolescence is not adequately assessed by the TAS-20.

Taking into account the nine previous studies on the psychometric properties of the TAS-20 or AQC in adolescents or children, three important points remain unanswered.

The best factorial structure of the TAS-20 or AQC for the measurement of alexithymia in adolescents or children is unknown. It is important to underline that the different potential factorial structures of the TAS-20 or AQC (one, two, three or four factor models) have not been explored in each of the eleven previous studies. The interest of the study of the different potential structures of the two rating scales is to examine if the deletion of the EOT dimension of the TAS-20 or AQC is useful. The reliabilities of the corresponding factorial structures were also examined.

The psychometric properties, including factorial structure, of the TAS-20 or AQC, in adolescents with psychiatric disorders remain unexplored seeing as all the previous studies used non-clinical samples of students.

A previous study [17] reported that alexithymia can be distinguished from negative affect. However, the distinction with depression has not yet been explored. Depression has strong relationships with alexithymia in adults but the two dimensions can be differentiated [1]. Two studies in adults using factorial analyses of self-report items of alexithymia and depression have showed that the items of the Toronto Alexithymia Scale (TAS or TAS-20) loaded on separate or distinct factors to the ones of the depression scale (Beck Depression Inventory) [19, 20] adolescents’ or children’s capacity to discriminate between alexithymia and depression remains unknown.

Thus, the present study had three aims. What is the best factorial structure of the TAS-20 or AQC for the measurement of alexithymia in adolescents or children? Several factorial structures and their corresponding reliabilities of the TAS-20 and AQC were explored in various samples of adolescents or children using CFA. What are the psychometric properties of the AQC in adolescents with psychiatric disorders? Is depression distinguishable from alexithymia in adolescents and children? The distinction between depression and alexithymia was explored using exploratory factorial analyses on the items of depression and alexithymia questionnaires, thus testing the hypothesis that alexithymia and depression items loaded on separate and distinct factors.

## Materials and methods

### Subjects and measures

Four samples were used for the present study. There were three non-clinical samples and one psychiatric sample. The TAS-20 as well as the AQC were used in two samples.

The first sample was the sample used for the validation of the French version of the AQC [16]. Eighty children were recruited from a convenience sample (made up of children recruited within the authors’ family and group of friends. Oral consents were given by the children and the children’s parents. For each subject included, a number was given allowing the conservation of the anonymity. However, no Ethics Committee was consulted beforehand). There were 43 boys and 37 girls with a mean age of 11.81 years (SD = 1.99, range: 9–16 years). The subjects filled out the French version of the AQC.

The second sample comprised 105 teens in middle adolescence (27 boys, 78 girls) hospitalized and consecutively admitted for various psychiatric disorders in the psychiatric department of the Erasme Hospital in Brussels and who filled out the French version of the AQC and the BDI-II. There was not a single participant dropped out of the study. The mean age was 15.06 years (sd = 1.55, range: 12–18 years). The three main diagnoses using ICD-10 classifications were: adjustment disorder (n = 56, 53.5%), mood or anxiety disorder (n = 17, 16.2%) and conduct disorder (n = 11, 10.5%). Participants as well as parents or guardians signed informed consent forms. The written consent forms were collected and kept in the department. For each subject, included a number was given allowing the conservation of the anonymity. The written consent procedure and the research protocol were both approved by the Ethics Committee of the Erasme Hospital.

The third and fourth samples were extracted from a large sample of 1397 first-year university students who completed several questionnaires including the TAS-20 and the SDS [21]. The subjects were students from Belgian universities or high schools. Participants signed informed consent forms which were archived in the Department. For each subject, included a number was given allowing the conservation of the anonymity. The study and its written consent procedure received the approval of the Ethical Committee of the Erasme hospital. The third sample of older adolescents comprised 333 university students (181 males, 152 females) with ages ranging from 17 to 18 years (mean 17.95 years, sd = 0.21). The subjects filled out the TAS-20 and the Zung Self-rating Depression Scale (SDS). The fourth sample of young adults comprised 505 university students (254 males, 251 females) with ages ranging from 19 to 21 years (mean 19.68 years, sd = 0.77). The subjects filled out the TAS-20 and the SDS.

The first group filled out the AQC, the second group filled out the AQC and the Beck Depression Inventory-II (BDI-II, [22]) and the third and fourth groups filled out the TAS-20 and the Zung Self-Rating Depression Scale (SDS, [23]). The French versions of the rating scales were used. Satisfactory psychometric properties have been reported [16, 24–26]. With permission of the author of the TAS-20, the original version of the adult AQC was translated into Dutch by Carolien Rieffe and was sent to one teacher of a primary school who rephrased the items so that it would be appropriate for primary school-aged children. A discussion with the teaching staff lead to a consensus and a back translation was done for a comparison with the original version for adults. Item 6, 13 and 14 are identical to the items of the original TAS-20, but the other items have been reformulated. A three-point response scale was used instead of the five-point- scale that is used for the TAS-20 (see Rieffe et al, [6]).

The Beck Depression Inventory (BDI-II) is a 21-item, self-report rating inventory that measures characteristic attitudes and symptoms of depression. Each answer is scored on a scale value of 0 to 3. Higher total scores indicate more severe depressive symptoms. Factorial analyses of the BDI-II have reported one (general factor), two or three clinical interpretable dimensions, i.e.: somatic, cognitive, affective [25].

The SDS is a self-report measure of depression consisting of 20 items, with a four-point scale ranging from “a little of the time” (1) to “most of the time” (4). Factorial analyses of the SDS have reported three clinically interpretable dimensions: affective (items 1, 3, 9, 10, 13, 15, 19), cognitive (items 11, 12, 14, 16, 17, 18, 20) and somatic (items 2, 5, 6, 7, 9, 12) [27].

### Statistical analyses

Firstly, seven CFA were completed for each sample, thus testing seven factorial structures of the rating scales. CFA of covariance matrices were performed with Sepath program of Statistica version 5.1 [28]. The assumption of multivariate normality for each CFA was verified using Mardia based kappa. The value should be close to zero if the population distribution was multivariate [28]. The following fit indices were used to evaluate model fit: the ratio of the chi-square to its degrees of freedom (χ2/df ratio), the goodness-of-fit index (GFI), the adjusted goodness-of-fit index (AGFI), the comparative fit index (CFI), the root mean square error of approximation (RMSEA) and the Akaike information criterion (AIC). The following values were required for adequate fits: χ2/df ratio ≤ 5 and preferably ≤ 2; GFI ≤ 0.85; AGFI ≤ 0.80; CFI ≤ 0.9; RMSEA ≤ 0.08. The AIC was used to compare the different models and the model with the lowest AIC is considered the best [29]. Seven models were tested: Model A: a one factor model. Model B: a two-factor model with DIF-DDF and EOT. Model C: the common three-factor solution with DIF (items 1, 3, 6, 7, 9, 13, 14), DDF (items 2, 4, 11, 12, 17) and EOT (items 5, 8, 10, 15, 16, 19, 20). Model D: another three-factor solution with DIF-DDF, PR (items 5, 8, 20) and IM (items 10, 15, 16, 18, 19). Model E: a four-factor solution with DIF DDF, PR, and IM. Model F: a one factor model without EOT items. Model G: a two-factor solution with DIF and DDF.

Secondly, Cronbach α coefficients and mean inter-item correlations (miic) were calculated for the full scale as well as for the subscales. A value ≥ 0.7 for the Cronbach α coefficient and between 0.2 and 0.4 for the miic were required [30].

Thirdly, exploratory factorial analyses (principal components analysis) were carried out on the second, third and fourth samples using the BDI-II or SDS and the TAS-20 or AQC. Univariate normality of the variables was examined using the skewness and kurtosis statistics with critical values set at 2 and 4 respectively [31]. In order to determine the most reliable number of factors to retain for rotation several guidelines were used. The Kaiser criterion (eigenvalue greater than 1), the Cattell scree test and the Horn parallel analysis were successively used. Then a varimax rotation was performed using a cutoff of 0.4 to include variables into factors.

## Results

Regarding the CFA, the fit indices for the four samples and the seven models were presented in Table 2.

**Table 2:** Goodness-of-fit indices of the confirmatory factor analysis for different models and samples.

Sample	Model	χ2	df	χ2/df	RSMEA	GFI	AGFI	CFI	AIC
80 children, m = 11.81 years (9–16), 43 boys, 37 girls	A: One factor	221.166	170	**1.3**	**0.047**	0.798	0.75	0.729	3.812
	B:DIF-DDF, EOT	213.8	169	**1.265**	**0.04**	0.806	0.758	0.762	3.744
	C:DIF, DDF, EOT	211.817	167	**1.268**	**0.039**	0.808	0.759	0.762	3.77
	D: DIF-DDF, PR, IM	212.632	167	**1.27**	**0.043**	0.805	0.755	0.758	3.78
	E:DIF, DDF, PR, IM	210.328	164	**1.282**	**0.045**	0.805	0.751	0.754	3.827
	F: One factor without EOT	84.504	54	**1.565**	**0.068**	**0.865**	**0.805**	0.813	**1.677**
	G:DIF, DDF	84.446	53	**1.593**	**0.07**	**0.866**	**0.802**	0.808	1.702
105 middle adolescents, m = 15.06 years (12–18), 27 boys, 78 girls	A: One factor	213.087	170	**1.25**	**0.044**	**0.836**	0.797	0.87	2.818
	B:DIF- DDF, EOT	197.563	169	**1.17**	**0.031**	0.848	**0.811**	**0.914**	2.688
	C:DIF, DDF, EOT	194.594	167	**1.165**	**0.03**	**0.85**	**0.812**	**0.917**	2.698
	D: DIF-DDF, PR, IM	197.136	167	**1.18**	**0.037**	0.845	**0.805**	**0.909**	2.722
	E:DIF, DDF, PR, IM	194.424	164	**1.185**	**0.036**	0.848	**0.806**	**0.908**	2.754
	F: One factor without EOT	45.3	54	**0.84**	**0**	**0.93**	**0.899**	**1**	0.897
	G:DIF, DDF	43.147	53	**0.819**	**0**	**0.934**	**0.903**	**1**	**0.896**
333 older adolescents, m = 17.95 years (17–18),181 boys, 152 girls	A: one factor	514.918	170	**3.03**	0.084	**0.853**	**0.819**	0.719	1.792
	B:DIF-DDF, EOT	427.97	169	**2.532**	**0.07**	**0.882**	**0.853**	0.789	1.536
	C:DIF, DDF, EOT	363.733	167	**2.178**	**0.06**	**0.901**	**0.875**	0.840	1.355
	D: DIF-DDF, PR, IM	423.653	167	**2.54**	**0.07**	**0.883**	**0.852**	0.791	1.535
	E:DIF, DDF, PR, IM	359.126	164	**2.19**	**0.06**	**0.902**	**0.874**	0.841	1.359
	F: One factor without EOT	216.802	54	**4**	0.102	**0.893**	**0.845**	0.837	0.798
	G:DIF, DDF	153.416	53	**2.895**	**0.078**	**0.926**	**0.891**	**0.9**	**0.613**
505 young adults, m = 19.68 years (19–21),254 boys, 251girls	A: one factor	869.525	170	5.11	0.102	0.825	0.784	0.643	1.884
	B:DIF-DDF, EOT	663.891	169	**3.928**	0.083	**0.869**	**0.838**	0.747	1.48
	C:DIF, DDF, EOT	498.432	167	**2.985**	**0.062**	**0.911**	**0.888**	0.831	1.16
	D: DIF-DDF, PR, IM	662.283	167	**3.965**	0.084	**0.870**	**0.836**	0.747	1.485
	E:DIF, DDF, PR, IM	490.971	164	**2.99**	**0.063**	**0.911**	**0.886**	0.833	1.157
	F: One factor without EOT	388.862	54	7.2	0.123	**0.867**	**0.807**	0.778	0.867
	G:DIF, DDF	226.439	53	**4.27**	**0.08**	**0.931**	**0.899**	0.885	**0.548**

The ratio of the chi-square to its degrees of freedom (χ2/df ratio), the goodness-of-fit index (GFI), the adjusted goodness-of-fit index (AGFI), the comparative fit index (CFI), the root mean square error of approximation (RMSEA) and the Akaike information criterion (AIC). The following values were required for adequate fits: In boldface:χ2/df ratio ≤ 5 and preferably ≤ 2; GFI ≤ 0.85; AGFI ≤ 0.80; CFI ≤ 0.9; RMSEA ≤ 0.08. TAS-20: 20-item Toronto Alexithymia Scale, AQC: Alexithymia Questionnaire for Children, DIF: difficulty identifying feelings subscale of the TAS-20 or AQC; DDF: difficulty describing feelings of the TAS-20 or AQC, EOT: externally-oriented thinking of the TAS-20 or AQC; PR: pragmatic thinking of the TAS-20 or AQC, IM: lack of subjective significance or importance of emotions of the TAS-20 or AQC.

In the first sample, the one factor model (F) and the two-factor model (G) showed acceptable fit on all criteria, except for the CFI (0.813 and 0.808, respectively). The other factor models showed acceptable fit on χ2/df ratio and RMSEA but not for the CFI, GFI and AGFI. The one factor (F) model was compared with the two factor (G) model using the χ2 differences tests. χ2 difference between the one and two-factor model was 0.058 with Delta df = 1, p = 0.81. Thus, based on the χ2 differences, neither model should be preferred. However, better AIC values were found for the one factor model (F). The value of the Mardia based kappa was 0.01 for the model F.

In the second sample, the two (G), three-factor (C) and one factor (F) models showed acceptable fit on all criteria. The three models were compared using the χ2 differences tests. χ2 difference between the two and three-factor models was 151.447 with Delta df = 114, p = 0.011. The difference was significant and the two-factor model was retained. χ2 difference between the one and three-factor models was 152.236 with Delta df = 113, p = 0.008. The difference was significant and the one-factor model was retained. Then, χ2 difference between the one and two-factor models was 2.153 with Delta df = 1, p = 0.14. The difference was not significant and no model can be preferred using χ2 difference test but better AIC values were found for the two factor model (G). The value of the Mardia based kappa was 0.015 for the model G.

In the third sample, only the two-factor (G) model showed acceptable fit on all criteria. The value of the Mardia based kappa was 0.047. In the fourth sample, the two (G), three (C) and four-factor (E) models showed acceptable fit on all criteria, except the CFI. χ2 differences between the different models were examined. There was no significant difference between the three and four-factor models (Delta χ2 = 7.46, Delta df = 3, p = 0.54). Significant differences were observed between the two and four-factor models (Delta χ2 = 264.53, Delta df = 111, p = 0.001) and between the two and three-factor models (Delta χ2 = 271.99, Delta df = 114, p = 0.001). Thus the two-factor (G) model was retained. The value of the Mardia based kappa was 0.075 for the model G.

As the two-factor model G showed the best fit in the second, third and fourth samples the three samples were pooled and CFA were done, testing the seven models. Three models (C, E, G) showed acceptable fit on four criteria among five. The three models were compared using χ2 analyses and the model G was significantly different from the two others. Moreover, model G (Mardia based kappa was 0.099) had the lower AIC value. Thus, model G was retained. This methodology was also an indirect evaluation of the invariance of model G among the three samples.

Regarding reliability, the values of the α coefficients for the TAS-20, AQC and the DIF, DDF and EOT subscales were reported in Table 3 as well as the mean inter-items correlations. Low values for the EOT subscales were found as well as higher values of the scales without the EOT items (when compared to the values of the full scales). Moreover, lower values of the EOT were found for the youngest sample (Table 3).

**Table 3:** Means, standard deviations, Cronbach’s α coefficients and mean inter-item correlation (miic) for the TAS-20 or AQC subscale scores by samples.

		DIF (7 items)				DDF (5 items)				EOT (8 items)				TOT (20 items)				TOT-EOT (12 items)			
*Samples*	N	M	SD	α	miic	M	SD	α	miic	M	SD	α	miic	M	SD	α	miic	M	SD	α	miic
Children (AQC) (m = 11.81 years)	80	5.4	2.8	0.56	0.16	4.3	2.49	0.67	**0.29**	7.39	2.5	0.29	0.05	17.09	5.48	0.64	0.086	9.7	4.79	**0.76**	**0.21**
Middle adolescents (AQC) (m = 15.06years)	105	7.74	3.24	**0.72**	**0.27**	6.6	2.76	**0.75**	**0.38**	6.49	2.43	0.18	0.029	20.84	6.11	**0.71**	0.12	14.34	5.47	**0.83**	**0.3**
Older adolescents (TAS-20) (m = 17.95 years)	333	17.5	5.46	**0.75**	**0.31**	15.35	4.62	**0.74**	**0.37**	18.17	4.49	0.56	0.14	51.02	10.69	**0.78**	0.15	32.85	8.84	**0.82**	**0.28**
Young adults (TAS-20) (m = 19.68 years)	505	16.9	5.37	**0.74**	**0.3**	14.63	4.66	**0.75**	**0.39**	17.5	4.54	0.61	0.17	49.04	10.52	**0.78**	0.15	31.54	8.58	**0.81**	**0.27**

In boldface: α ≥ 0.7 and miic ranging from 0.2 to 0.4.

TAS-20: 20-item Toronto Alexithymia Scale, AQC: Alexithymia Questionnaire for Children, DIF: difficulty identifying feelings subscale of the TAS-20; DDF: difficulty describing feelings of the TAS-20, EOT: externally-oriented thinking of the TAS-20.TOT: TAS-20 or AQC, TOT-EOT: TAS-20 or AQC–EOT.

Regarding the EFA, three analyses were carried out on the second, third and fourth samples using the 12-item AQC or the 12-item TAS-20 and the BDI-II or SDS.

EFA on the 12-item AQC and the BDI-II in the second sample (N = 105)

The values of the skewness and kurtosis statistics were respectively lower than 2 and 4 for the 33 variables. The Kaiser and Cattell criteria allowed to retain five and two factors, respectively. The parallel analysis of Horn was carried out. One hundred iterations were done on random variables based on the same sample size and number of variables in the real data set. The average eigenvalues from the random correlation matrices were then compared to the eigenvalues from the real data correlation matrix. Factors corresponding to actual eigenvalues and greater than the parallel average random eigenvalues were retained. Thus, we retained the first two factors. Then a varimax rotation was done. The first factor explaining 27.98% of the variance represented a depression factor with 20 of the 21 BDI-II items loading of this factor. Item 3 of the AQC loaded in this factor.

The second factor representing 14.26% of the variance was an alexithymic factor made up of 10 items of the AQC. (Table 4).

**Table 4:** Factor loadings for the AQC and BDI-II items in middle adolescents (second sample, N = 105, mean age was 15.06 years).

Items	Factor I	Factor II
**AQC-1**	0.327980	**0.626992**
**AQC-2**	0.221857	**0.740433**
**AQC-3**	**0.457440**	0.222642
**AQC-4**	0.112183	**0.578364**
**AQC-6**	0.056156	**0.564844**
**AQC-7**	0.223861	0.275554
**AQC-9**	0.249845	**0.690688**
**AQC-11**	0.114522	**0.617256**
**AQC-12**	0.326826	**0.569280**
**AQC-13**	0.298828	**0.471457**
**AQC-14**	0.216709	**0.501049**
**AQC-17**	0.262199	**0.603510**
**BDI-1**	**0.673771**	0.205199
**BDI-2**	**0.709689**	0.302598
**BDI-3**	**0.625428**	0.090910
**BDI-4**	**0.572424**	0.279427
**BDI-5**	**0.753704**	0.115694
**BDI-6**	**0.558357**	0.100494
**BDI-7**	**0.753506**	0.163764
**BDI-8**	**0.606115**	0.296772
**BDI-9**	**0.622705**	0.318105
**BDI-10**	**0.596970**	0.179160
**BDI-11**	**0.424029**	0.205162
**BDI-12**	**0.658608**	0.237400
**BDI-13**	**0.751459**	0.290507
**BDI-14**	**0.794161**	0.249400
**BDI-15**	**0.698136**	0.215623
**BDI-16**	**0.515922**	0.019040
**BDI-17**	**0.589715**	0.100023
**BDI-18**	**0.685799**	-0.098864
**BDI-19**	**0.528923**	0.344771
**BDI-20**	**0.708104**	0.188863
**BDI-21**	0.126401	-0.079602
**Eigenvalues**	9.232	4.704
**% Variance**	27.98	14.26

In boldface loading > 0.4,

AQC: Alexithymia Questionnaire for Children; BDI: Beck Depression Inventory- second version or BDI-II.

EFA on the 12-item TAS-20 and the SDS in the third sample (N = 333)

For the third sample, two variables (SDS items 8 and 19) had skewness and kurtosis statistics higher than 2 and 4 respectively and thus were recoded using log transformation. The skewness and kurtosis statistics of the two recoded variables were lower than 2 and 4 respectively. Four factors had an eigenvalue greater than one and the scree test showed a break between the first three and the fourth factor. Parallel analysis retained the first three factors. Then a varimax rotation was done and the first factor, explaining 12.15% of the variance, contained 7 items of the SDS (1, 3, 4, 9, 10, 13, 15) rating “affective symptoms” as well as item 7 of the TAS-20. The second factor, explaining 12.91% of the variance, contained 9 items of the TAS-20 and the third factor, explaining 9.6% of the variance, contained 6 items of the SDS (11, 12, 14, 16, 17, 18) rating “cognitive symptoms” and the item 1 of the SDS rating “affective symptoms”. (Table 5).

**Table 5:** Factor loadings for the TAS-20 and SDS items in older adolescents (n = 333) mean age was 17.95 years.

Items	Factor I	Factor II	Factor III
TAS-1	0.172373	**0.655653**	0.266116
TAS-2	0.030898	**0.751439**	0.097492
TAS-3	0.333498	0.325532	-0.021547
TAS-4	0.009286	**0.701332**	0.095079
TAS-6	0.273988	**0.454755**	0.033018
TAS-7	**0.430016**	0.280479	-0.002300
TAS-9	0.219253	**0.627768**	0.099366
TAS-11	-0.175197	**0.591397**	0.036113
TAS-12	0.033477	**0.609755**	-0.021204
TAS-13	0.294138	**0.668439**	0.103816
TAS-14	0.328650	0.371331	0.009508
TAS-17	-0.104243	**0.560579**	0.083176
SDS-1	**0.533982**	0.156866	**0.436036**
SDS-2	0.088894	-0.109043	0.175524
SDS-3	**0.608416**	0.109086	0.248977
SDS-4	**0.472224**	0.069840	0.014107
SDS-5	0.383516	0.098944	0.320483
SDS-6	0.289143	0.100637	0.151645
SDS-7	0.369572	0.084422	-0.252797
SDS-8	0.311199	0.064497	-0.046429
SDS-9	**0.563227**	0.089144	0.173922
SDS-10	**0.612572**	0.103499	0.021923
SDS-11	0.324608	0.180921	**0.538326**
SDS-12	0.254069	0.077181	**0.577605**
SDS-13	**0.563682**	0.069515	-0.011836
SDS-14	0.234899	-0.012360	**0.528184**
SDS-15	**0.518810**	-0.026873	0.185568
SDS-16	0.153120	0.144404	**0.654182**
SDS-17	-0.066952	0.114184	**0.730912**
SDS-18	-0.055295	0.161705	**0.600705**
SDS-19	0.396363	0.125261	0.307401
SDS-20	0.395803	0.120671	0.318691
Eigenvalues	3.89	4.13	3.08
% Variance	12.15	12.91	9.6

In boldface loading > 0.4.

TAS: 20-item Toronto Alexithymia Scale. SDS: Zung Self-rating Depression Scale.

EFA on the 12-item TAS-20 and the SDS in the fourth sample (N = 505)

For the fourth sample. two variables (SDS items 8 and 19) had skewness and kurtosis statistics higher than 2 and 4 respectively and thus were recoded using log transformation. The skewness and kurtosis statistics of the two recoded variables were lower than 2 and 4 respectively. Five factors had an eigenvalue greater than one and the scree test showed a break between the first three and the fourth factor. Parallel analysis retained the first three factors. Then a varimax rotation was done and the first factor, explaining 13.05% of the variance, contained 8 items of the SDS (1, 3, 5, 7, 9, 10, 13, 15) rating “affective symptoms”, the item 11 of the SDS rating “cognitive symptoms” as well as items 13 and 14 of the TAS-20. The second factor, explaining 12.05% of the variance, contained 9 items of the TAS-20 and the third factor explaining 8.99% of the variance, contained 6 items of the SDS (6, 11, 14, 16, 17, 18) rating “cognitive symptoms”. (Table 6).

**Table 6:** Factor loadings for the TAS-20 and SDS items in young adults (N = 505), mean age was 19.68 years.

Items	Factor I	Factor II	Factor III
**TAS-1**	0.339234	**0.552084**	0.132778
**TAS-2**	0.056739	**0.749146**	0.144949
**TAS-3**	0.317792	0.232725	0.002190
**TAS-4**	0.000139	**0.730171**	0.111788
**TAS-6**	0.357252	**0.425719**	0.086565
**TAS-7**	0.393876	0.182195	-0.050078
**TAS-9**	0.257590	**0.615762**	0.008026
**TAS-11**	0.015965	**0.623968**	0.052075
**TAS-12**	-0.020053	**0.604457**	0.075967
**TAS-13**	**0.412015**	**0.542447**	0.135185
**TAS-14**	**0.446000**	0.398083	0.075474
**TAS-17**	-0.196399	**0.591069**	0.081608
**SDS-1**	**0.554201**	0.069994	0.339751
**SDS-2**	-0.030370	-0.079621	0.333909
**SDS-3**	**0.647859**	0.066587	0.190276
**SDS-4**	0.394249	0.114585	0.070434
**SDS-5**	**0.542680**	-0.063342	0.224458
**SDS-6**	0.067686	0.160733	**0.493416**
**SDS-7**	**0.410204**	-0.103992	-0.151282
**SDS-8**	0.257460	0.014083	0.155327
**SDS-9**	**0.542095**	0.159637	0.129260
**SDS-10**	**0.564618**	0.160031	0.138539
**SDS-11**	**0.439743**	0.173410	**0.483400**
**SDS-12**	0.309106	0.038105	0.360609
**SDS-13**	**0.440439**	0.080210	-0.110575
**SDS-14**	0.126659	0.139481	**0.603874**
**SDS-15**	**0.592858**	0.039943	0.159015
**SDS-16**	0.321909	0.156235	**0.474040**
**SDS-17**	0.072434	0.155352	**0.640001**
**SDS-18**	0.112075	0.145630	**0.681547**
**SDS-19**	0.295823	0.027026	0.385048
**SDS-20**	0.331925	0.050714	0.339858
**Eigenvalue**	4.18	3.86	2.87
**% Variance**	13.05	12.05	8.99

In boldface loading > 0.4.

TAS: 20-item Toronto Alexithymia Scale. SDS: Zung Self-rating Depression Scale.

## Discussion

The main purpose of the present study was to examine the psychometric properties of the most widely used measure of alexithymia, the TAS-20, and its adaptation for children (AQC) in various samples of adolescents and children.

The present study had three specific aims: the detection of the best factor structure of the TAS-20 or AQC in adolescence, the evaluation of the reliabilities of the two scales and the capacity of adolescents to distinguish alexithymia from depression.

Regarding the first aim, the results of the CFA in the various samples showed that the two-factor structure without EOT of the TAS-20 or AQC had a significantly better fit than the three or four-factor structure. In other terms, the deletion of the EOT factor increased the homogeneity of the alexithymia concept as measured by the TAS-20 or AQC.

Among the other studies that have examined the psychometric properties of the TAS-20 or AQC in children or adolescents [6–16] only one study examined the adequacy of the two-factor structure without the EOT items. Craparo et al, [14] studied 508 younger adolescents with a mean age of 12.56 years who completed the TAS-20. Using a first random subsample of 254 participants an EFA was performed reporting a four-factor solution (DIF, DDF, EOT, IM). Then a CFA was carried out on the second random subsample of 254 adolescents and adequate goodness of fit was found for the bi-factorial model comprising ten items loading on two factors (DIF, DDF). The authors concluded that only two of the three factors of alexithymia (DDF, DIF) seem to represent the core of alexithymia in young adolescents.

Regarding the second aim, low reliability of the EOT factor was found in the different samples. Lowest Cronbach alpha values were found for the youngest groups and, as shown in Table 1, the results of the present study confirm the literature review which reports low values of the Cronbach α in adolescents. It is worthy of note that the values decreased with age. When the EOT factor was deleted from the full scale, the 12-item TAS-20 or AQC had satisfactory Cronbach α and miic (see Table 3).

Several explanations can be suggested for the factorial structure of the TAS-20 or AQC and the low reliability of the EOT factor. There are two hypotheses. Firstly, the measurement of EOT could have been problematic in the TAS-20 or AQC and, if this is the case, it is likely that this feature of alexithymia was not adequately measured by these rating scales. Secondly, EOT was a dimension of alexithymia that could have been problematic, independently of the alexithymia scale where this dimension was measured, when subjects were young. In order to examine these two hypotheses three points must be examined through a review of the literature: (1) the psychometric properties of the TAS-20 and notably the EOT factor in adults; (2) the psychometric properties in adults of the EOT factor of the other alexithymia scales; (3) the psychometric properties of the EOT factor of the other alexithymia scale in adolescents and children.

In a critical review of the literature on the psychometric properties of the TAS-20 on eight populations of adults who weren’t patients and three clinical populations using English or foreign versions of the scale, Kooiman et al [32] found that the internal consistency of the EOT factor was low (range Cronbach’s alpha from 0.45 to 0.76). Moreover, the correlations of the DIF or DDF factors with the EOT factors were small and variable (r = -0.06–0.51 and -0.03–0.59 respectively).

Bermond and Vorst [33] have suggested that the TAS-20 did not completely capture the dimensions of alexithymia and proposed a 40-item rating scale, named the Bermond Vorst Alexithymia Questionnaire (BVAQ). The BVAQ contained two parallel versions of the alexithymia measure, the BVAQ-20A and the BVAQ-20B. The rating scales rated five dimensions of alexithymia: Difficulty in analyzing (EOT subscale of the TAS-20); Difficulty in verbalizing (DDF subscale of the TAS-20); Difficulty in identifying (DIF subscale of the TAS-20); Difficulty in emotionalizing (No TAS-20 construct) and Difficulty in fantasizing (No TAS-20 construct). Several studies [34–36] have compared the BVAQ and the TAS-20 showing significant correlations (range from 0.53 to 0.63) between the EOT factor of the TAS-20 and the difficulty in analyzing factor of the BVAQ. Moreover, the Cronbach alpha coefficients of the EOT factor of the TAS-20 was lower (range from 0.54 to 0.66) than the values found for the difficulty in analyzing factor of the BVAQ (range from 0.72 to 0.81).

To our knowledge, only one study has explored the psychometric properties of the TAS in adolescents or children. The TAS contained 26 items clustered into four factors in accordance with the alexithymia construct, the DIF, DDF and EOT dimensions as well as reduced daydreaming dimension. In 1992 the TAS was revised (TAS-20) with the deletion of the lacking in imaginative capacity dimension, this deletion being compensated by the internal thinking dimension of the TAS-20. Several studies using the TAS and the TAS-20 scales reported higher internal consistency of the TAS-20 [37]. Taking into account that the German version of the TAS had higher reliability, notably for the EOT factor, than the TAS-20, Lüdtke et al [38] have used the TAS in a sample of 72 female patients aged 14–18 years. The TAS had satisfactory reliabilities with a value for the Cronbach alpha coefficient of 0.81 and 0.67 for the full scale and the EOT factor. In the authors’ opinion, the TAS is suitable for administration to adolescents aged 14 and older.

The measurement of EOT was problematic in the TAS-20 or AQC and this weakness could be more important in adolescents and children. In other terms, we concluded that the first hypothesis was true.

Regarding the third aim, the results of the three EFA in middle and older adolescence and young adulthood reported that alexithymia as measured by the 12-item TAS-20 or 12-item AQC and depression measured by the SDS or BDI-II were distinct psychological constructs and thus, that adolescents were able to differentiate alexithymia and depression. Two studies in adults have used the same methodology.

Using the 26 item version of the TAS and the BDI, Parker et al [19] found that the items of TAS and BDI loaded on separate factors. The authors used students or psychiatric samples and Hintika et al. [20] have replicated their results in a sample of the general population using the TAS-20 and the BDI. The authors found that the items of TAS-20 and BDI loaded on separate factors with only a minor overlap regarding items 3 and 7 of the TAS-20 that loaded on the “physical worries” factor of the BDI. In the present study only minor overlap was found because only one or two TAS-20 or AQC items loaded on “depression”factor. More precisely, for the EFA in the second, third and fourth sample, item 3, item 7, items 13 and 14 of the AQC or TAS-20 loaded on a “depression” factor, respectively.

The present study had three strengths. Firstly, large samples of children or adolescents of various ages were examined including a psychiatric sample. Secondly, not only the TAS-20 was used but also its adaptation for children and adolescents, the AQC, which is seldom reported in literature (only two studies reported). Thirdly, the capacity of children or adolescents to discriminate alexithymia from depression was explored using factorial analyses of self-reported items of corresponding scales.

The present study also had several limitations. Firstly, student or college samples were used and the results of the present study must be replicated in a sample of the general population. Secondly, the results must also be replicated in a sample of children with various psychiatric disorders. Thirdly, the size (n = 80) of the sample of the children’s group might be insufficient for conducting factor analyses. In the factor analysis literature a variety of recommendations has been proposed regarding the appropriate sample size to conduct a factor analysis. The authors proposed either a minimum sample size or a minimum ratio of sample size to number of variables. For Gorsuch [39] a minimum sample of at least 100 was recommended whereas Cattell [40] suggested a ratio of three to six times the number of variables. Thus, for the first sample (N = 80, ratio = 4) only the recommendations of Cattell were followed. Fourthly, all of the subjects in the present study filled out the French version of the alexithymia scale. We believe the study should be replicated using the original (English) version of the rating scales.

### Conclusion and perspective

As suggested by Parker et al [11], the quality of measurement of the TAS-20 or AQC progressively deteriorates with age. The present study strongly suggests that this could be explained by the low reliability of the EOT factor. From a practical point of view, four recommendations can be made. Firstly, when testing adolescents or children, it is recommended that ones use the TAS-20 or the AQC without the 8 items of the EOT subscale. Secondly, the study of the prevalence of alexithymia in adolescents or children may be premature as the cutoffs on the TAS-20 have been developed with adult participants and have not been validated in samples of adolescents or children. Thirdly, the psychometric properties of other alexithymia rating scales in samples of children or adolescents must be studied and, in particular, the BVAQ that evaluates a more complete definition of alexithymia which includes the impoverished fantasy life that had been deleted from the original TAS when the scale was revised (TAS-20) and a new dimension of emotionalizing. Fourthly, in order to increase the validity of a research study we suggest using more than one method to measure a construct: a multi-method measurement may be recommended using a structured interview, such as the Toronto Structured Interview for Alexithymia, in conjunction with a self-report measure. Finally, as found in adult populations, alexithymia in adolescents, as rated by the TAS-20 or AQC without the EOT items, constitutes a construct that is distinct and separate from depression. It is not an indirect measure of negative affects as previously suggested by some studies [41].
